# Towards the Use of Adsorption Methods for the Removal of Purines from Beer

**DOI:** 10.3390/molecules26216460

**Published:** 2021-10-26

**Authors:** Catarina Almeida, Márcia C. Neves, Mara G. Freire

**Affiliations:** Department of Chemistry, CICECO-Aveiro Institute of Materials, University of Aveiro, 3810-193 Aveiro, Portugal; ac.almeida@ua.pt (C.A.); mcneves@ua.pt (M.C.N.)

**Keywords:** beer, purine compounds, uric acid, hyperuricemia, gout, enzymatic methods, biological methods, adsorption, purine removal

## Abstract

Beer corresponds to a fermented alcoholic beverage composed of several components, including purine compounds. These molecules, when ingested by humans, can be catabolized into uric acid, contributing to uric acid’s level increase in serum, which may lead to hyperuricemia and gout. To assure a proper management of this disease, physicians recommend restrictive dietary measures, particularly by avoiding the consumption of beer. Therefore, it is of relevance to develop efficient methods to remove purine compounds from alcoholic beverages such as beer. In this review, we provide an introduction on fermented alcoholic beverages, with emphasis on beer, as well as its purine compounds and their role in uric acid metabolism in the human body in relation to hyperuricemia and gout development. The several reported enzymatic, biological and adsorption methods envisaging purine compounds’ removal are then reviewed. Some enzymatic and biological methods present drawbacks, which can be overcome by adsorption methods. Within adsorption methods, adsorbent materials, such as activated carbon or charcoal, have been reported and applied to beer or wort samples, showing an excellent capacity for adsorbing and removing purine compounds. Although the main topic of this review is on the removal of purine compounds from beer, other studies involving other matrices rather than beer or wort that are rich in purines are included, since they provide relevant clues on designing efficient removal processes. By ensuring the selective removal of purine compounds from this beverage, beer can be taken by hyperuricemic and gouty patients, avoiding restrictive dietary measures, while decreasing the related healthcare economic burden.

## 1. Fermented Alcoholic Beverages

The consumption of alcoholic beverages dates from the time of ancient civilizations, and they have been referred to as playing an important societal role [1,2]. The first evidence of alcohol use refers to the Paleolithic period, occurring by the intake of grapes exposed to a fermentation event, i.e., by the exposure of the native micro flora to the sun [3]. During many years, the manufacture of alcoholic beverages was accomplished in this spontaneous form; however, with the emergence of agriculture practices, the fermentation process was introduced, initially in Mesopotamia, approximately in 8000 BC, having spread to other civilizations [3,4,5]. Afterwards, with the industrial revolution and the appearance of microbiology, the fermentation methods started to be industrialized and better known [6]. Along the years, a significant development has been verified in this field, with a continuous increase in the corresponding market [3,7].

Alcoholic beverages are described as liquids designated for human consumption that result from a fermentation process, in which fermentable sugars are catabolized into ethanol, as well as a variety of other compounds that can determine their particular attributes [3,4]. Depending on the production methods, raw materials and ethanol content, several groups of alcoholic beverages can be distinguished, for instance, beer and wine as fermented alcoholic beverages, and distilled liquors or spirits [8,9]. Regarding their processing, fermented alcoholic beverages are obtained only through a fermentation process, while distilled liquors are produced by the distillation of the ethanol produced during fermentation [8]. Regarding the starting materials, cereal grains are used to produce beers and whiskies, fruits to obtain wines and brandies, and molasses and sugar cane to produce rum [10]. The alcohol content varies according to the type of alcoholic beverage; for instance, beer exhibits a content of 4–8%, wines present 5–13% of alcohol and distilled spirits have a content of 40–60% [8,9].

Fermented alcoholic beverages play an important cultural and economic role in the society [2,5]. The key process inherent to their production is alcoholic fermentation, an event that occurs under oxygen deprivation conditions in which, through the action of yeast, fermentable sugars derived from the raw materials are converted into ethanol and carbon dioxide [10,11]. Generally, yeasts from the *Saccharomyces* genus are used in the manufacture of alcoholic beverages, particularly *Saccharomyces cerevisiae* strains; however, other microorganisms can be employed leading to different final products [10]. Therefore, an initial stage focused on yeast growth and reproduction takes place, in which yeasts are provided with the suitable nutrients, followed by their uptake across the cellular plasma membrane and their assimilation [10]. Within the variety of essential nutrients, numerous macronutrients can be recognized, including sugars (e.g., glucose, maltose, sucrose, lactose and fructose), organic nitrogen compounds (e.g., amino acids and small peptides), inorganic nitrogen (e.g., ammonium salts), oxygen, several anions and cations (e.g., sulfur, potassium, magnesium, and phosphorus), micronutrients (e.g., calcium, manganese, copper, iron and zinc) and various growth factors (e.g., vitamins, nucleotides, nucleosides, purines and pyrimidines bases, fatty acids, amino acids and sterols) [10]. Afterwards, during the yeast fermentative metabolism phase, sugar molecules derived from the hydrolysis of carbohydrates and related constituents present in raw materials can be used as carbon sources, as well as electron acceptors and donors [10]. Typically, glucose is the most preferred carbon source; however, it is not often available in the industrial fermentation media, leading to other alternatives (e.g., maltose, etc.) to be used, varying according to the desired final product [10,11]. Besides ethanol and carbon dioxide, which are considered the main fermentation metabolites, several other by-products such as higher alcohols, esters, organic acids, aldehydes, etc., can be obtained, many of them responsible for the development of specific sensory properties of the final product [5,10]. Fermented products have aroused significant interest and, nowadays, numerous production techniques, raw materials and microorganisms are used, allowing the production of a wide variety of beers [6,12].

## 2. Beer, a Fermented Alcoholic Beverage

Globally considered as one of the oldest and most important alcoholic beverages, beer corresponds to the third most popular drink among consumers, after coffee and tea [12,13,14]. According to the World Health Organization (WHO), beer dominates the second position on the scale of the most consumed alcoholic beverages worldwide, representing 34.3% of the total alcohol consumed, being preceded solely by spirits (44.8%) [15]. The designation of beer derives from the Latin term “biber”, which means “drink”, and it is defined as a fermented beverage obtained from starch and flavored with hops [16,17,18].

**Figure 1:**
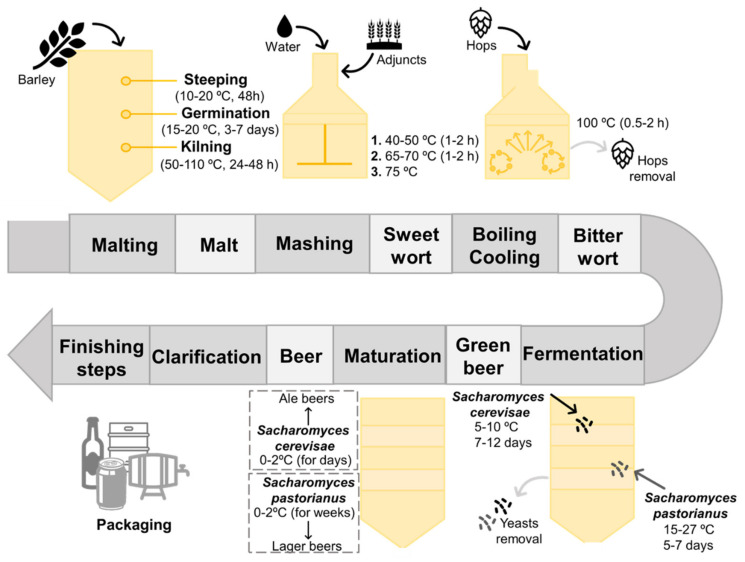
Schematic overview of the brewing process.

Up to date, there are four base natural products needed to produce beer, namely malt, yeasts, hops and water [16,18]. The set of processes involved in the production of beer is termed brewing, being summarized in Figure 1 [14,18]. Malting corresponds to the first phase of brewing, comprising a set of stages, including steeping, germination and kilning, which lead to the production of malt from raw cereal grains [13,19,20]. Barley (Hordeum vulgare) is the most frequently used cereal in beer production, since it can grow easily, even in adverse climate conditions; can be easily converted into malt; displays a suitable protein concentration (10–12%) required to yeast growth and foam production; and exhibits a significant carbohydrate content of 78–83%, of which 63–65% correspond to starch (one of the main sources of fermentable sugars used by yeasts during their fermentative metabolism) [17,18,21]. Barley grains are initially steeped in aerated water conditions to increase their humidity, and then they are germinated at temperatures fluctuating from 15 to 20 °C, for 3 to 7 days [13,21]. During this stage, several biochemical modifications occur in the grains, resulting on the synthesis of a variety of enzymes that are responsible for the breakdown of endosperm structural components, starch and proteins [17,22]. After germination and subsequent removal of the sprouts, a kilning stage takes place to yield dry malted grains, in which controlled high temperatures are employed to reduce the water content of the grains without inactivating the enzymes, along with the occurrence of several Maillard reactions that are responsible for the generation of color and flavor-related compounds [13,17,21,22,23]. In sum, the resultant cereal malt consists in a mixture of different components with a crucial importance in the next steps of brewing, such as saccharides, proteins, free amino nitrogen (FAN), enzymes and purine compounds, being considered the major contributors to the sensorial characteristics of the finished beer [21,23].

During the second phase of brewing, termed mashing, the previous obtained malt is mixed with boiling water, followed by a resting period under regulated temperatures to enhance the activity of enzymes [14,16,17,21,22]. Despite the popularity of barley, other substrates, acting as malt adjuncts, can be also introduced during this stage, for instance rice, sorghum, corn, wheat, sugars, syrups, etc., contributing not only to supply starch and sugar, but also to the development of specific attributes and leading to a wide diversity of beers [17,21]. The temperature is initially maintained between 40 and 50 °C to enhance α-amylases, followed by an increment to temperatures between 65 and 70 °C to stimulate ß-amylases [21]. At this time, we succeed in the hydrolyzation of ß-glucans by ß-glucanases, the hydrolyzation of proteins into their amino acids and peptides through the action of proteases, and the hydrolyzation of starch by α-amylases and ß-amylases into fermentable sugars, with maltose being the most abundant [16,21,22,24]. Afterwards, the temperature is increased to at least 75 °C to inactivate the former enzymes, followed by a filtration step, resulting in a sweet wort that is defined as a complex mixture of fermentable sugars and other nutrients necessary for yeasts metabolism [16,18,20,21]. The resultant wort is then transferred to a heating container (also known as brewing kettle) and hops are added, followed by a boiling stage for 1 to 2 h [14,16,21,22,25]. Hops corresponds to the flower of the *Humulus lupulus* plant, contributing to the aroma, flavor and stability of the finished beer due to the presence of several essential oils and resins (e.g., α-acids or humulone, ß-acids or lupulone, and tannins), some of them responsible for the aroma and bitterness profile of beer [21,22,25,26]. During the boiling step, several events take place: (1) microorganisms and enzymes that remained active at the end of the mashing phase are killed and inactivated, respectively; (2) certain undesirable compounds are precipitated and removed; (3) resins and essential oils are extracted from hops, allowing the conversion of α-acids into their soluble form to improve the bitterness character, concentrate the flavor, and sterilize the wort; (4) color-related attributes are developed; (5) the remaining water is evaporated; and (6) the resultant wort is concentrated [21,22,25]. At the end of this phase, wort is separated from the flavor agents, followed by a cooling step, resulting in a bitter wort that is transferred to a fermentation vessel [16,21]. The next step in brewing is focused on the wort inoculation with yeasts, initially under aeration conditions to promote their growth, followed by an alcoholic fermentation process in which ethanol and carbon dioxide are formed [10,14,16,21,22]. As stressed before, other by-products can be obtained from the yeast metabolism; however, they are obtained at lower concentrations [10,27].

**Figure 2:**
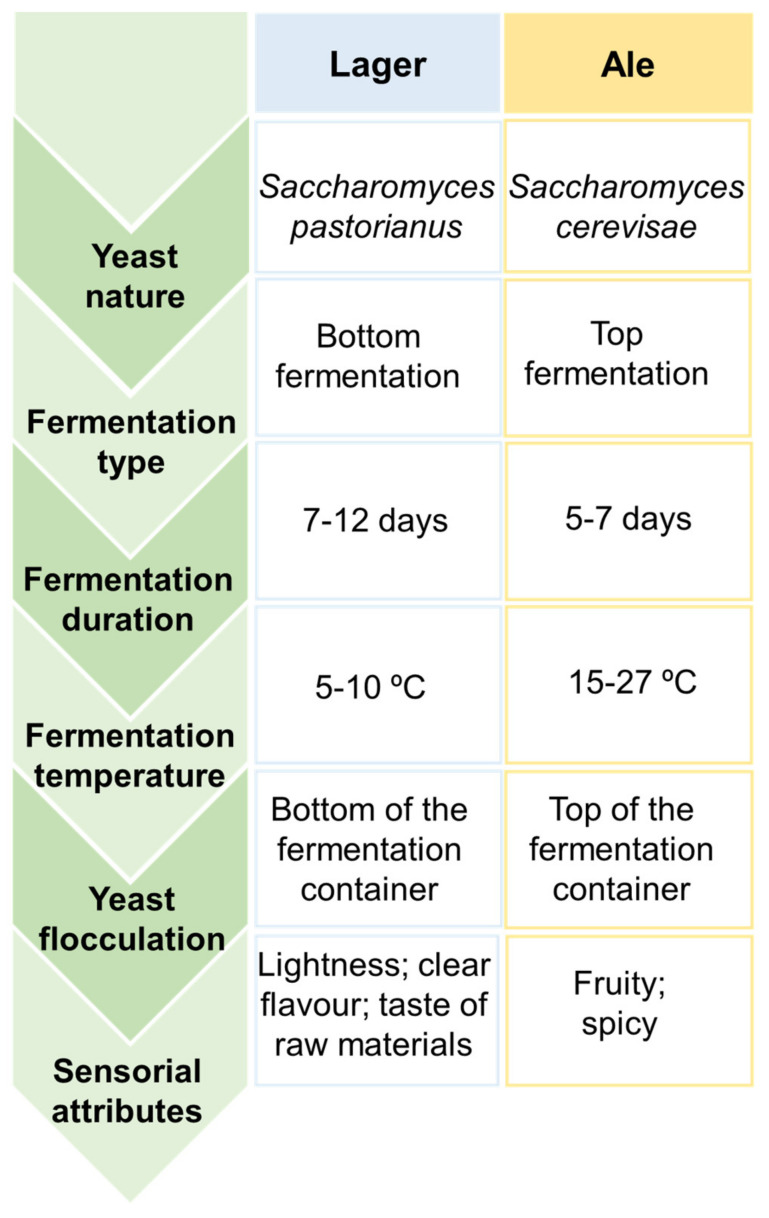
Main differences between Lager and Ale beers.

Depending on the nature of yeasts that remained at the end of the later process and the temperatures applied, two types of fermentation can be distinguished, particularly top and bottom-fermentations, that result on the rise of two main classes of beer, known as Ale and Lager, with the latter being the most common [10,13,14,16,26]. The main differences between these two beer styles are summarized in Figure 2.

Top-fermentations are characterized by being a rapid process performed by top-fermenting yeasts, such as strains of *Saccharomyces cerevisiae*, under high temperatures ranging from 18 to 27 °C for 5–7 days [16,25,26]. This kind of yeast has a predisposition to rise to the surface of the wort, adhere to carbon dioxide molecules, and subsequently produce foam. In contrast, bottom-fermentations are known by being a slow process accomplished by bottom-fermenting yeasts, such as strains of *Saccharomyces carlsbergensis* (also known as *Saccharomyces pastorianus*) and *Saccharomyces uvarum*, that act under low temperatures ranging from 6–15 °C for 7–12 days [21,25]. At the end of the fermentation process, these latter yeasts tend to flocculate and sink to the bottom of the wort. Therefore, top-fermentation are involved in Ale beers production, which is characterized by fruity and spicy attributes given by specific aroma-related compounds (e.g., esters, volatile phenols, etc.), while bottom-fermentation is associated to Lager beers manufacture, which is characterized by a lightness and cleaner flavor [13,16,25].

After fermentation takes place, yeasts are removed, resulting in a non-consumable beer, known as green beer [16]. Therefore, a maturation step must occur, under temperatures ranging from 0 to 2 °C for several days; however, the time required for maturation depends on the intended beer (e.g., lager beers takes longer times to be aged than ale beers) [16,21]. This stage allows not only the removal of undesirable compounds but also the development of the final flavor, color and other attributes. Certain brewery industries include also a conditioning step, under temperatures of −1 °C, for at least 3 days, allowing the clarification through removal of the remaining yeast, proteins, and other suspended solids [21,25]. The ultimate phase of brewing is focused on the proper preparation of beer to be commercialized. Depending on the desired final product, distinct processing steps, some of them optional, are applied: (1) addition of proteases to counteract haze generation by residual proteins, during storage under refrigeration temperatures; (2) clarification and filtration to remove the remaining suspended solids; and (3) adjustment of carbon dioxide level (0.45–0.52%) by a carbonation method that implies the addition of this molecule to the final product or, as an alternative, the addition of a fresh yeasted wort to allow the occurrence of a secondary fermentation [21]. The finished beer can be packaged into cans, bottles, kegs and/or barrels. However, before being placed on the market, certain beers, particularly those that require a longer conservation period, such as canned and bottled beers, are subjected to an additional treatment, usually pasteurization, to improve their shelf-life, while others require a higher period of inoculation with yeasts to enhance a secondary fermentation process and, consequently, to endorse the production of by-products responsible for specific flavors [16,21,22].

The chemical analysis of beer is crucial to determine and guarantee its quality and safety, but also to understand its nutritional aspects and organoleptic attributes [18]. Apart from water, which is the major constituent representing around 90% of the final product, beer presents other molecules, namely carbohydrates, ethanol and carbon dioxide [17,28]. Despite most of the sugar present in wort being fermented into ethanol during brewing, some non-fermentable carbohydrates remain at the end of the process [17]. Carbohydrates represent 3.3 to 4.4% of the final product, of which 75–80% correspond to dextrins, 20–30% to mono and oligosaccharides and 5–8% to pentosans [28]. Ethanol is considered the most important alcohol in beer that also acts as a flavor and sweetness enhancer, being found at concentrations that vary according to the type of beverage [17,18]. For instance, it can range from 20,000 to 80,000 mg L^−1^, with a percentage of alcohol per volume variable from 0.05% in alcohol-free beers to 12.5% in British Thomas Hardly Ale (the strongest beer worldwide) [18]. Carbon dioxide is typically found at concentration ranging from 3.5 to 4.5 g·L^−1^; however, certain types of beers, such as those that are bottle conditioned, can exhibit higher contents [17]. Carbon dioxide is a result of yeast metabolism, which impacts foam generation and the flavor profile of the finished product since it assures the proper delivery of volatile compounds into the headspace of beers. Along with these major compounds, beer encompasses other circa 800 organic and non-organic compounds, being present in smaller amounts. Some of them are derived from raw materials and do not suffer any alteration during brewing, while others are produced as by-products due to yeasts metabolism. The main constituents of beer, their contents and sources are summarized in Table 1.

Inorganic salts, derived from malt and/or water used during brewing, can be found at concentrations ranging from 500 to 2000 mg L^−1^, comprising cations, such as magnesium and sodium; anions, such as chloride and sulfate; and trace metals [17,18,28]. Depending on their nature and concentration, these elements can produce negative or positive effects on the sensorial profile of the finished beer, influencing the taste and clarity properties [17,18]. Several alcohols can also be present, such as higher alcohols (e.g., 3-methylbutanol, 2-methylbutanol, 2-methyl-propanol, propanol and ß-phenylethanol) at concentrations ranging from 60 to 100 mg L^−1^; contents higher than 100 mg L^−1^ can led to negative effects on flavor and need to be regulated [17]. Some phenolic alcohols can also be found, being derived from the breakdown of malt and from hops polyphenols. These are responsible for the texture and harshness, as well as glycerol derived from yeast metabolism, which play an important role on taste, enhancing smoothness and mouthfeel. Organic acids (e.g., acetic acid, lactic acid and succinic acid), which are derived from yeast metabolism through deamination reactions of the amino acids present in wort, can also be found at concentration ranging from 200 to 500 mg L^−1^ [17,28]. These compounds are responsible for the relative acidity of beer (normal pH of 4.0–5.0; however, some beers have a pH around 3.0). Small amounts of vitamins can be found, namely complex B vitamins (e.g., biotin, inositol, pantothenic acid, etc.), that act as growth factors assimilable by yeast to ensure their performance, justifying their low contents on beer [17,18]. Esters and aldehydes, generated through esterification and dehydrogenation reactions of alcohols, respectively, are also present [17]. Esters (e.g., ethyl esters) can be found at concentrations ranging from 60 to 80 mg L^−1^ and are related to the fruity and floral aromas, while aldehydes (e.g., acetaldehyde) are present with contents ranging from 10 to 20 mg L^−1^, being associated with the characteristic young flavor of green beer. Beer additionally presents hop derivatives, such as iso-α-acids, at concentrations ranging from 10 to 100 mg·mL^−1^ [18]. They are considered the primary flavor constituents, after ethanol and carbon dioxide, being responsible for the beer bitterness, foam stabilization and inhibition of microbial growth. Sulphur compounds (e.g., sulfur dioxide, hydrogen sulfite, etc.) derived from malt, hop and yeast metabolism, are present at concentrations ranging from 1 to 10 mg L^−1^, being responsible for the overall flavor profile of beer, while allowing the monitorization of the processing operations involved in brewing [17,18]. Finally, beer possess a wide range of nitrogenous compounds, found with a content ranging from 300 to 1000 mg L^−1^, of which 80–85% are derived from malt and the remaining from yeast metabolism [17,29]. This comprises amino acids, peptides, polypeptides, amines, nucleic acid and derivatives, heterocyclic compounds, and particularly metabolites that result from nucleic acids catabolism, such as purine and pyrimidine compounds. During the mashing phase, proteins and peptides are catabolized into their amino acids, resulting in high contents of nitrogenous compounds available to the yeast growth. Therefore, most of the amino acids present in the finished beer correspond to those that were not assimilated or used by yeast to produce higher alcohols [17]. Moreover, during malting and mashing phases of brewing, nucleic acids are enzymatically catabolized into their respective purine and pyrimidine nucleotides, nucleosides and free bases, contributing to their appearance in the finished beer [17,18,29]. Little is known regarding pyrimidine compounds in beer; however, the presence of purine compounds in this beverage is well known and documented [17].

### 2.1. Purine Compounds in Beer

**Figure 3:**
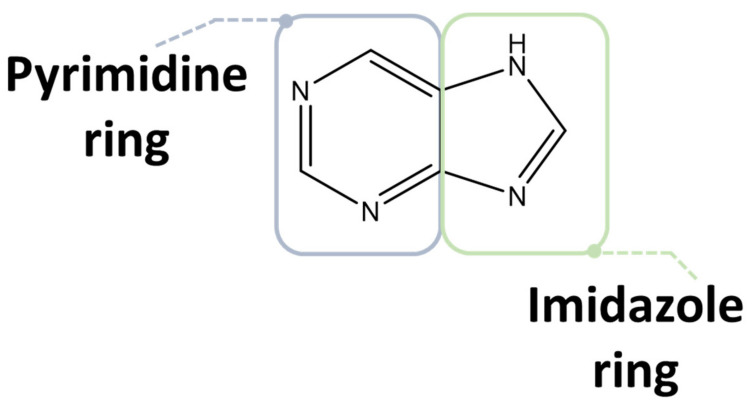
Double-ring structured purine compound.

Purine compounds are of vital importance in all living organisms. Purines, which refer to the substituted purines and respective tautomers, are heterocyclic aromatic compounds formed by the fusion of a pyrimidine and an imidazole ring, as shown in Figure 3 [30,31].

**Figure 4:**
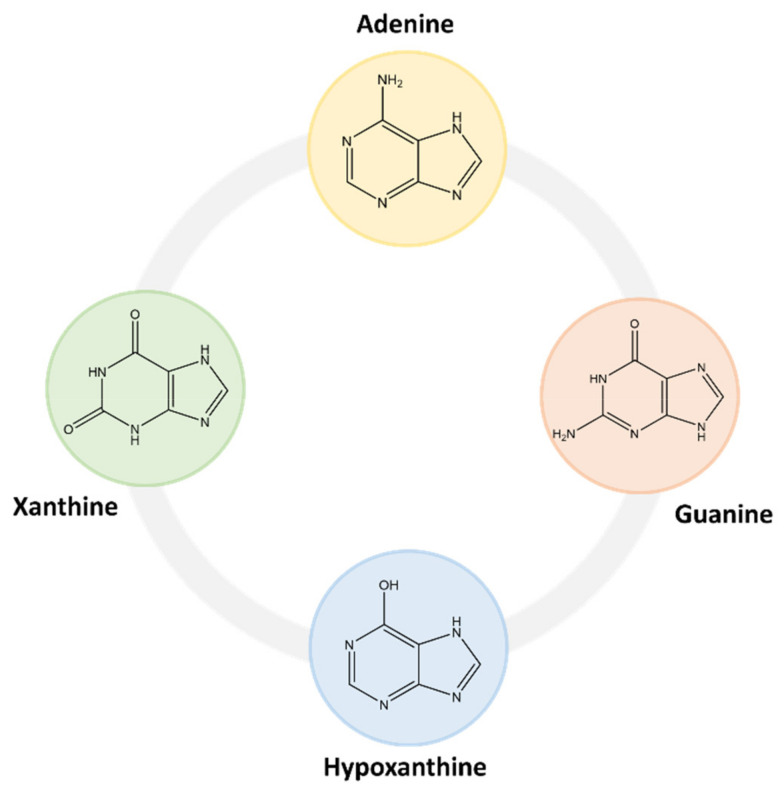
Universe of purine compounds and their chemical structures.

Within this class of compounds are included bases, nucleosides and nucleotides (Table 2), and some examples are adenine, guanine, hypoxanthine and xanthine (Figure 4) [31,32]. 

In mammals, purines are more commonly expressed into nucleic acids, for instance ribonucleic and deoxyribonucleic acids (RNA and DNA, respectively), which contain the two purine bases adenine and guanine, several single-molecule nucleotides such as ATP, ADP, adenosine monophosphate (AMP) and cyclic adenosine monophosphate (cAMP) and, to a lesser extent, guanosine triphosphate (GTP) and cyclic guanosine monophosphate (cGMP) [30].

Purines are recognized for their important biological roles in different organisms in addition to mammals, such as bacteria, fungi, viruses and plants [31]. They are involved in the storage and transmission of genetic information, signal transduction and translation as GTP, cAMP and cGMP, energy supply as ATP to keep many cellular reactions functioning, and have a role in the energy metabolism of several compounds, such as NAD, NADPH and coenzymes (e.g., coenzyme Q) [30,34]. Purines may also act as neurotransmitters as it may be described by the interaction of adenosine with certain receptors, resulting in the modulation of the immune and inflammatory systems [35].

**Figure 5:**
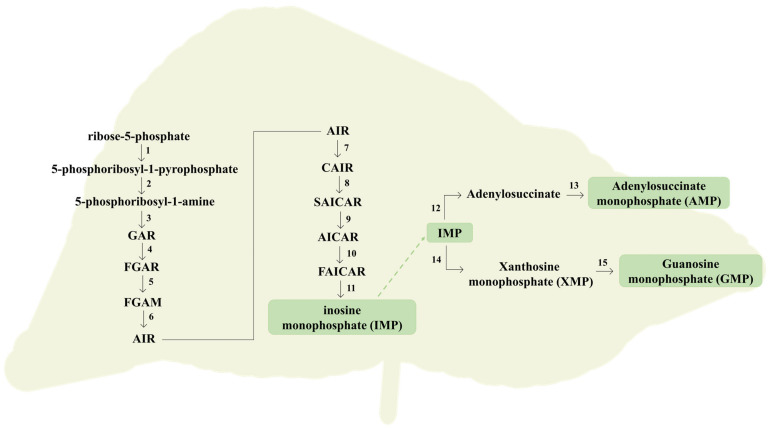
Overview of the purine biosynthesis through de novo synthetic pathway. The enzymes involved are represented as numbers: (1) PRPP synthase, (2) glutamine-PRPP amidotransferase, (3) GAR synthetase, (4) GAR transformylase, (5) FGAR amidotransferase, (6) AIR synthetase, (7) AIR carboxylase, (8) SAICAR synthetase, (9) SAICAR lyase, (10) AICAR transformylase, (11) IMP synthase, (12) adenylosuccinate synthetase, (13) adenylosuccinate lyase, (14) IMP dehydrogenase and (15) XMP-glutamine amidotransferase. ATP, adenosine triphosphate; AMP, adenosine monophosphate; PPi, pyrophosphate; ADP, adenosine diphosphate; GAR, glycinamide ribonucleotide; FGAR, formylglycinamide ribonucleotide; FGAM, formylglycinamidine ribonucleotide; AIR, 5-aminoimidazole ribonucleotide; CAIR, carboxyaminoimidazole ribonucleotide; SAICAR, N-succinyl-5-aminoimidazole-4-carboxyamide ribonucleotide; AICAR, 5-aminoimidazole-4-carboxyamide ribonucleotide; FAICAR, N-formylaminoimidazole-4-carboxamide ribonucleotide.

To surpass the essential needs for their growth and survival, cells require a balanced supply of purine compounds [34]. In humans, they can be obtained exogenously by dietary intake or endogenously produced through two metabolic pathways known as de novo synthesis and salvage pathway [34,36]. De novo synthesis occurs mainly in the liver, consisting in a multi-step synthetic route, where several small starting molecules are combined to form the characteristic double ring of a purine [36]. The overview of the de novo synthesis of purines is summarized in Figure 5. Primarily, pyrophosphate (PPi) provided by ATP, and ribose-5-phosphate experience a condensation, catalyzed by the enzyme phosphoribosylpyrophosphate synthase (PRPPS), to form 5-phosphoribosyl-1-pyrophosphate (PRPP) [30,36]. Subsequently, an amino group from glutamine is added to PRPP, which is converted into 5-phosphoribosyl-1-amine, in a reaction catalyzed by glutamine phosphoribosyl amidotransferase (APRT). This sequence of reactions is often considered as rate limiting and the involved enzymes are exposed to a feedback inhibition promoted by purines, which slow the biosynthesis route when the purine reservations are considered sufficient [30,37]. Thereafter, an amino group from glycine is added and formylated by N10-formyltetrahydrofolate, followed by nitrogen contribution by glutamine, yielding 5-aminoimidazole ribonucleotide, the characteristic 5-membered imidazole ring of purines [37]. Then, a carboxylation catalyzed by 5-aminoimidazole ribonucleotide (AIR) carboxylase occurs, converting the latter metabolite into carboxyaminoimidazole ribonucleotide. Finally, an amino group donation from aspartate with fumarate release, followed by carbon contribution by N10-formyltetrahydrofolate, contributes to yield the characteristic 6-membered pyrimidine ring of purines. The first formed intermediate with a complete purine double ring corresponds to inosine monophosphate (IMP) [36,37].

The resultant IMP can be converted into AMP through a sequence of reactions: (1) formation of adenylosuccinate from IMP catalyzed by adenylosuccinate synthetase, using aspartate and GTP as a source of amino group and energy, respectively; and (2) conversion of adenylosuccinate into AMP, catalyzed by adenylosuccinate lyase [30,37]. On the other hand, IMP can be also converted into GMP, involving an oxidation of IMP to XMP catalyzed by IMP dehydrogenase, followed by the formation of GMP catalyzed by XMP-glutamine amidotransferase, using glutamine and ATP as a source of an amino group and energy, respectively. The resultant AMP and GMP can be further phosphorylated into higher energy molecules, such as ATP and GTP [30]. From a global perspective, this synthetic pathway is more complex since it is dependent of energy supply, requiring the consumption of multiple molecules of ATP to increase the purine turnover [30,34]. Once generated by de novo synthesis, nucleotides are inserted into the purine pool to further be used in metabolic processes [36]. Given the energy expenditure involved in the de novo synthesis, a less complex mechanism occurs to surpass this limitation, termed the salvage pathway [30]. This strategy is focused on the recycling or salvage of free purine bases and nucleosides, constantly release in cells due to metabolic turnover and dietary intake before their catabolism takes place, to synthetized nucleotides [36,37]. Initially, a phosphoribose is transferred from PRPP to the free bases hypoxanthine and guanine, yielding the respective nucleotides IMP and GMP, through a reaction catalyzed by hypoxanthine-guanine phosphoribosyltransferase (HGPPRT) [30,36]. The resulting nucleotides can be reintroduced in the purine pool [30]. Furthermore, a reaction between free adenine and PRPP, catalyzed by adenosine phosphoribosyltransferase, takes place, yielding the respective nucleotide AMP; however, most of the adenosine/adenine breakdown occurs through conversion to IMP [30,36]. In normal circumstances, the salvage pathway predominates over the de novo synthesis. Nevertheless, the latter route is considered particularly active in tissues with a higher rate of cell turnover [34].

Although purines are naturally synthetized in the human body, some of them, including nucleic acids, nucleotides, nucleosides and free bases, are exogenously obtained from diet through the ingestion of certain foods and beverages, being the major source of the daily purine load, including beer [30,38,39]. The main sources of purines in food products are nucleic acids existing as nucleoproteins, which are enzymatically catabolized by the action of 5′-nucleotidases and phosphatases to form their respective nucleosides and purine bases [32,38,40]. Subsequently, these two latter compounds are absorbed in the small intestine and utilized by the purine metabolic pathway [32,39,41].

**Figure 6:**
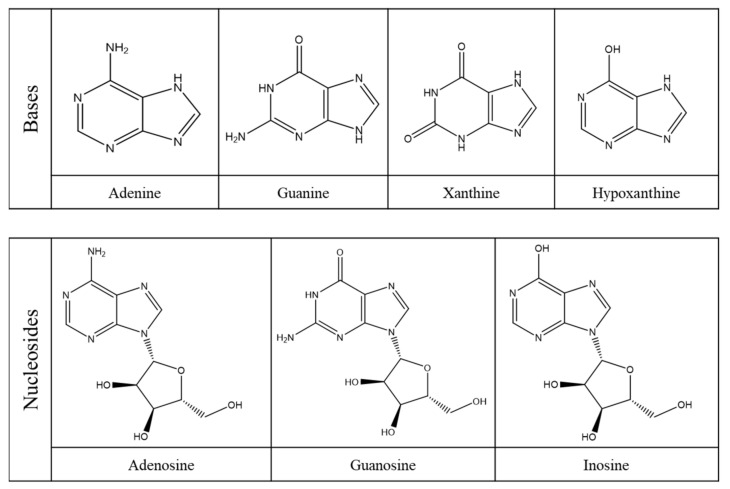
Most common purine nucleosides and bases found in beer.

There are comprehensive reviews, although few, showing the occurrence of purine compounds in a diversity of food products [39,41]. For instance, Wu et al. [41] reviewed the presence of four purine compounds (e.g., adenine, guanine, hypoxanthine and xanthine) in foods, alcoholic beverages and dietary supplements. Similarly, Kaneko et al. [39] reviewed the total and individual purine contents in numerous food products. From these works it can be pointed out that purine-rich foods are those that are cooked or processed, derived from animal or seafood sources, such as red, organ, lamb and pork meats; shellfish; vegetable-base foods, for instance, mushrooms, lentils, beans, legumes and peas; dairy products; vitamin C-rich foods; plant oils; and alcoholic beverages, with beer accounting for the highest purine content [31,41,42]. Most of the purines present in beer are derived from malt and consist mainly in nucleosides and bases [31,43]. Examples of the most found purines in beer are given in Figure 6. Free purine bases can be found at lower concentrations due to their absorption and metabolization by yeast during fermentation, while nucleosides do not change their content during brewing, since they are not assimilated by yeast [43]. Nucleotides and macromolecular nucleic acids can also be found, but in lower amounts.

There are some reports describing the quantification of purines in beer, many of them employing HPLC [32,33,38,44,45,46]; however, other analytical methods are also applied, such as Capillary Zone Electrophoresis (CZE) [47] and Micellar Electrokinetic Capillary Chromatography (MECK) [48]. Significant variations on purine contents among distinct beer samples were found in the majority of these studies, while highlighting guanosine as the most abundant purine.

Harris and Parsons [49], in 1956, were among the first investigators demonstrating the presence of the purine nucleosides adenosine and guanosine, and the purine bases adenine and guanine in wort, using paper chromatography. Gibson et al. [50] quantified several purines, including adenine, hypoxanthine, xanthine, adenosine and guanosine in seven British beers, comparing them to Guinness, cider, home-brewed beer, and lager beer. An average total purine nitrogen content of 22.2 mg L^−1^ was found in all British beers tested, in which 61% corresponded to guanosine. Similar results were obtained for Guinness, while slightly lower contents were registered in lager beer (17.7 mg/mg L^−1^ L; 40% guanosine), and negligible amounts were found in cider (0.4 mg L^−1^; guanosine not detected) and home-brewed beer (3.9 mg L^−1^; 46% guanosine). Overall, it was concluded that beer presents a higher purine content in comparison to other alcoholic beverages, with guanosine accounting for the most abundant purine compound. Yamamoto et al. [33] determined the contents of hypoxanthine, xanthine, inosine, adenosine, and guanosine in beer, describing as well guanosine (174 µmol L^−1^) as the most abundant purine compound. Klampfl et al. [47] quantified five purines (adenine, hypoxanthine, xanthine, adenosine and guanosine) in distinct types of beer. Variations on their contents were found between the investigated samples, which may be due to the differences in the raw materials and brewing processes involved. Moreover, it was concluded that beers with lower alcohol contents present lower purine concentrations and, similarly to the previously mentioned studies, guanosine was described as the most abundant (an average of 90 mg L^−1^) purine compound in all beers tested. Cortacero-Ramírez et al. [48] analyzed simultaneously 26 components, among them adenosine and adenine, in seven beer samples. Variations on their contents were found between samples, which also may be due to the use of distinct raw materials and brewing processes. The higher adenosine content (21.3 ± 1.9 mg L^−1^) was found in light beer, while the higher adenine content (3.0 ± 0.4 mg L^−1^) was found in classic I beer; nevertheless, adenine was not detected in four of the analyzed beers. Cortacero-Ramírez et al. [51] quantified multiple constituents, including adenosine, adenine, guanosine, and xanthine in beer of different origins. Xanthine and guanosine were found to be the most abundant purine compounds in all beers analyzed. Markelj et al. [52] quantified purine bases, namely xanthine, adenine and guanosine in biological fluids and several food samples, including alcoholic and non-alcoholic beers. The results showed similar total purine contents between the two types of beer (alcoholic beer: 533–664 µM; non-alcoholic beer: 638–678 µM), in which guanosine was the most abundant (alcoholic beer: 187–288 µM; non-alcoholic beer: 203–232 µM), followed by xanthine and adenine.

Some studies focused on the determination of purine contents in various alcoholic beverages are reported, highlighting beer as the most abundant in purine compounds [32,38,46]. The comparison of total purines content between alcoholic beverages are given in Table 3. For instance, Kaneko et al. [46] determined the concentration of four purines, namely adenine, guanine, hypoxanthine and xanthine in several alcoholic beverages (spirits, beers, and other liquors). Beer exhibited a higher purine content (1145.7–13.3 µmol L^−1^) in comparison to other alcoholic beverages tested, such as spirits (26.4–0.7 µmol L^−1^) and other liquors (sake: 110.4–82.1 µmol L^−1^; wine: 108.0–28.3 µmol L^−1^; shoku-shu: 818.3–537.4 µmol L^−1^; Ume Liqueur: 13.1 µmol L^−1^, and beer-flavored beverage: 157.4–73.5 µmol L^−1^). By analyzing the purines individually, it was concluded that guanine was predominant in beers. A bottle (350 mL) of regular beer exhibited a higher purine content (787–2031 µmol), comparing to a serving of low-malt beer (938–677 µmol), low-malt and low-purine beer (46 µmol), and a shot (40 mL) of spirits, such as whisky, brandy, and shoko-shu (10.6–0.3 µmol) [46]. Fukuuchi et al. [32] quantified purines in beer and beer-like beverages. A total purine content of more than 5.0 mg/100 mL was found in beer, with guanine accounting for the majority, while beer-like beverages exhibited lower purine concentrations of 3.6–0.13 mg/100 mL. It was also concluded that a can (350 mL) of beer presents a purine amount of 18.2–4.2 mg, whereas in beer-like beverages lower values (0.46–12.6 mg) were achieved [32]. Li et al. [38] determined the contents of guanine, adenine, hypoxanthine and xanthine in numerous Chinese domestic beers and other alcoholic beverages. The results showed that beer presents a higher total purine content (average content: 74.89 mg L^−1^) than other alcoholic beverages (Chinese spirits: 0.22 mg L^−1^; rice wine: 45.99 mg L^−1^, and wine: 29.67 mg L^−1^). It was also concluded that guanine is the most abundant purine in beer (19–80 mg L^−1^), followed by adenine (<30 mg L^−1^), while hypoxanthine and xanthine exhibited inferior contents (<20 mg L^−1^), excepting in one pale beer in which xanthine verified a concentration of 39.3 mg L^−1^ [38].

### 2.2. From Purine Compounds Catabolism to Hyperuricemia and Gout

**Figure 7:**
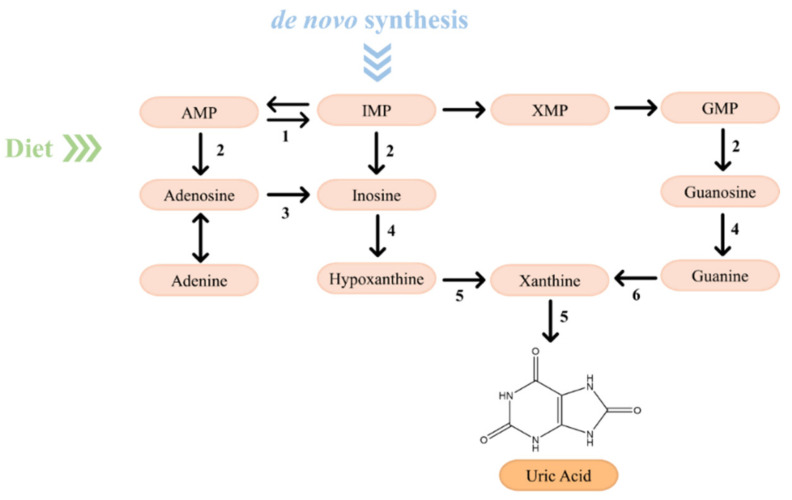
Overview of the purine catabolism into uric acid. The enzymes involved are represented by numbers: (1) AMP deaminase, (2) nucleotidase, (3) adenosine deaminase, (4) purine nucleoside phosphorylase (PNP), (5) xanthine oxidase and (6) guanine deaminase.

Purines, generated by de novo synthesis and salvage pathway or derived from dietary intake, are susceptible to enzymatic catabolism into uric acid to maintain their homeostasis [30,53,54]. Thus, approximately one-third of the uric acid is obtained by dietary intake, while the remaining two-thirds is endogenously produced by purine metabolism [55]. The purine catabolism (Figure 7) occurs mainly in the liver and involves two major key compounds, the monophosphate nucleotides IMP and GMP [30,53,56]. AMP is converted to inosine through two distinct mechanisms: (1) removal of an amino group of AMP, catalyzed by AMP deaminase, to form IMP, followed by a dephosphorylation promoted by a nucleotidase to generate inosine; and (2) removal of a phosphate group of AMP, catalyzed by a nucleotidase to form adenosine with, subsequently, deamination to generate inosine [30,53]. Simultaneously, IMP and GMP are converted into inosine and guanosine, respectively, through a reaction catalyzed by a nucleotidase [53]. Then, guanosine and inosine are converted into guanine and hypoxanthine, respectively, in a reaction catalyzed by a purine nucleoside phosphorylase (PNP). Afterwards, hypoxanthine is oxidized by xanthine oxidase (XO) and guanine is deaminated by guanine deaminase to both form xanthine which, in turn, is oxidized by XO into uric acid [30,53,56].

In most mammals, the purine catabolism does not cease with the formation of uric acid due to the presence of urate oxidase, more commonly known as uricase, which converts this latter metabolite into the more soluble compound allantoin [30,55,57]. However, humans and other primates are unable to perform it due to the lack of this enzyme, making uric acid, the end-product of the purine metabolism [53,55,58]. Uric acid (C_5_H_4_N_4_O_3_) is a heterocyclic aromatic compound with a molecular weight of 168 Da, constituted by a 2,6,8-trihydroxypurine existing as a keto-enol tautomerism (7,9-dihydro-1H-purine-2,6,8(3H)-trione) [53,58]. It is considered one of the most important nitrogenous compounds in several organisms, playing a crucial role in humans [59]. Due to its double bonds, uric acid exhibits a good antioxidant capability, accounting for approximately two-thirds (up to 60%) of the total plasma antioxidant capacity [53,56,59]. At physiological pH conditions, it is considered a weak acid with a pKa of 5.8 and circulates in plasma mostly in its monovalent sodium salt, known as urate [53,56]. It exhibits a low solubility in water (also in plasma) and generally, in human blood, its average concentration is close to the solubility limit of 6.8 mg dL^−1^. The reference interval of uric acid in human blood varies according to the gender, being 1.5–6.0 and 2.5–7.0 mg dL^−1^ in women and men, respectively [53]. However, when serum uric acid (SUA) is found at concentrations above its solubility limit, monosodium urate (MSU) crystals can be formed, contributing to hyperuricemia [53,58].

Hyperuricemia is a pathological condition characterized by an increase of serum uric acid levels above a specific threshold, which may lead to uric acid crystallization into MSU crystals, and further deposition in joints and surrounding tissues [53,55,57]. Hyperuricemia is generally defined by SUA levels ≥7.0 mg dL^−1^ and ≥6.0 mg dL^−1^ for adult men and women, respectively [53]. Its prevalence has gradually increased over the past years, with several epidemiological studies proving this trend [60,61,62]. For instance, the US National Health and Nutrition Examination Survey (NHANES) [61,63] revealed a significant increase of hyperuricemia prevalence from 19.1% in 1988–1994 to 21.5% in 2007/2008; however, according to the data available for 2009/2010, the prevalence remained practically unchanged (19.3%). Similarly, a representative study on the hyperuricemia prevalence in adult population of Italy, showed an increment from 85.4 per 1000 inhabitants in 2005 to 119.3 per 1000 inhabitants in 2009 [62]. Moreover, hyperuricemia prevalence in individuals of the coastal cities of Eastern China increased to 13.2% in 2004, in contrast to the values equal to zero registered in the 1980s [64].

Serum uric acid levels are determined by the balance between uric acid production and excretion [30,42]. Therefore, three main events are involved in hyperuricemia development: (1) uric acid overproduction and/or excessive intake, (2) impair uric acid excretion and (3) a combination of both [53,55,65]. Mechanisms should exist to ensure the efficient disposal of uric acid and, since humans are incapable to metabolize it, excretion processes play a crucial role in maintaining homeostasis [30]. Its excretion occurs mainly in the kidneys, representing approximately two-thirds, with the remaining one-third confined to the gastrointestinal tract, in which up to 70% is eliminated in urine and feces [53,55]. If the uric acid daily supply exceeds the excretion capacity, or if there is any impairment in its elimination, hyperuricemia can take place [55]. Hyperuricemia can also appear as a result of uric acid overproduction [53,55,57]. Therefore, diets rich in purines and fructose or exposure to lead seem to trigger the increase of uric acid levels [53,58]. Certain enzymatic deficiencies can also contribute to the increase of uric acid levels [30,42]. Moreover, an accelerated cellular turnover, visible in some malignancies and hematological and inflammatory diseases, contributes to uric acid overproduction [30]. Despite its multiple causes, impairments that lead to uric acid overproduction are responsible for only 10% of the hyperuricemic cases, while most of the patients present abnormalities in their renal uric acid excretion. Hyperuricemia is frequently related to the precursors of various comorbidities, contributing to a higher risk to develop them, and this includes metabolic diseases, such as type 2 diabetes mellitus [66] and metabolic syndrome [67]; hypertension [68]; cardiovascular illnesses, such as coronary artery disease [69], heart failure [70] and stroke [71]; pre-eclampsia [72]; and gout [73].

Gout is the most common form of inflammatory arthritis, a human systemic disease that is in a continuous globally rising, especially in the developed fraction of the world [42,55,74]. It results from the MSU crystals formation and deposition within joints and other tissues, such as soft tissues, occurring due to chronic hyperuricemia, which is defined as a persistent increment of SUA levels above a specific threshold [42,75,76]. Despite hyperuricemia being considered a prerequisite for gout development, it should be remarked that not all hyperuricemic subjects manifest this disease or even form uric acid crystals [42,56]. In the Hypertension Detection and Follow-Up Program, individuals with SUA levels ranging from 7.0 to 7.9 mg dL^−1^ were followed up for 14 years, showing that 12% of the participants developed gout [77]. SUA levels above 9.0 mg dL^−1^ have a higher prognostic role on the development of gout; however, these values are unusual, occurring in less than 20% of patients with chronic hyperuricemia [78]. These findings suggest that, although hyperuricemia is a required condition, alone it is not sufficient to develop gout and, therefore other factors may also contribute [42].

There is not a single estimate for a global gout prevalence since it varies widely according to the geographical background of populations, methodologies applied, and disease definitions used; however, a prevalence ranging from 1 to 4% of the general population is found [42,74,75]. According to epidemiological surveys published in recent years, it was suggested that gout prevalence and incidence is increasing worldwide, particularly in developed countries (Table 4) [74,75].

In fact, in an epidemiological examination conducted by the US National Health and Nutrition Examination Survey (NHANES), from 2007 to 2008, a gout prevalence of 3.9% was found in a representative sample of the US adult population [61]. Similarly, in a more recent study conducted by NHADES, using data from 2007 to 2016, it was concluded that gout prevalence remained constant [79]. In South Korea, it was estimated a gout prevalence of 3.49 per 1000 individuals in 2007, that increased to 7.58 per 1000 individuals in 2015, while predicting a further increment of 1.66% by 2025 [80]. In Australia, from 2013 to 2016, a gout prevalence of 1.6% was estimated [81], whereas, in the United Kingdom, it was estimated to be 2.49% in 2012, having suffered an increment of around 64% since 1997 [82]. In an epidemiological study conducted in Portugal from 2011 to 2013, involving 10,661 randomly selected adults, it was shown an estimate gout prevalence of 1.3% [83].

Gout progresses in four main stages, including asymptomatic hyperuricemia, acute gouty attack, intercritical period and chronic tophaceous gout [36,42,54]. The first phase is characterized by an absence of symptoms or signs, such as pain and swelling of the joints; however, SUA levels are above the reference threshold [36,42]. The acute gouty attacks are usually monoarthritic, being characterized by a sudden onset of pain and swelling of the joint that peaks within hours to severely inflamed joint with signs of inflammation, such as hotness, redness, tenderness, swelling and loss of function. The intercritical period corresponds to a remission phase that is characterized by an absence of symptoms and occurs when the previous acute attacks are resolved with a proper treatment, which takes place within hours to days. During this stage, crystals deposits may still be present; however, at lower levels, explaining the possibility of future gouty attacks [54]. If an adequate treatment has not been implemented, attacks become more frequent and severe [42]. Finally, when the disease is untreated, a chronic tophaceous gout stage takes place, which is characterized by an increment on the frequency of acute attacks overtime, accompanied by the development of a mass formed by the accumulated MSU crystals, known as tophus [36,42]. This stage corresponds to a manifestation of a chronic and uncontrolled gout and the time needed to form a palpable tophus, after the first gouty attacks takes place, varies from 3 to 40 years. Tophus may lead to joint destruction and deformity, as also to bone erosions, when the growing extent of the mass extrapolates to the bone [42].

Gout risk factors include age, gender, geographic background, race and genetic predisposition, and lifestyle-related factors, such as diabetes and dietary and exercising habits [84,85,86,87]. Although SUA levels are normally distributed in the general population, it can be recognized a significant difference between genders, in which men are more likely to exhibit higher values [84]. This could be the reason why gout prevalence in men are typically 2–6 folds higher than in women [42,84]. Using data from 2007 to 2016, the prevalence in men was estimated to be 5.2%, while, in women, a prevalence of 2.7% was registered [79]. Gout prevalence increases with age [87,88], with lower values (0.4%) found in individuals of 20–29 years, and a higher prevalence (12.6%) in individuals with 80 years or older [61]. Geographical and racial distribution also have an impact on gout occurrence, being more prevalent within ethnic minorities [88].

Finally, lifestyle-associated factors play a fundamental role in the regulation of SUA levels and, consequently, have an impact in gout development [42,84,89]. In this are included poor dietary habits, alcohol consumption, an increased incidence of diabetes and metabolic syndrome and the ingestion of purine-rich foods and beverages [42,74]. The effect of alcoholic beverages consumption on the development of hyperuricemia and gout depends on the amount of alcohol and on the type of beverage consumed, with studies reporting beer as the alcoholic beverage that possess the higher risk [42]. For instance, Nishioka et al. [90] studied the influence of congeners from various alcoholic beverages in SUA levels of healthy male individuals that consumed 0.8 mL of ethanol equivalents per kilogram of body weight. The SUA levels before and after one-hour drinking, in comparison with the baseline, were 5.4 ± 0.2 and 5.2 ± 0.2 mg dL^−1^ for whisky, 5.3 ± 0.3 and 5.2 ± 0.2 mg dL^−1^ for shochu and 5.1 ± 0.3 and 5.8 ± 0.3 mg dL^−1^ for beer. These findings suggest that even a moderate drinking of beer induces an increase in SUA levels, and, when compared with other alcoholic beverages, beer offers a higher risk. Nakamura et al. [91] compared the risk of incident hyperuricemia between individuals who drank only beer and non-drinkers, and a positive dose–response association between beer intake and the risk of hyperuricemia was verified. In addition to its ethanol content, the considerable purine concentration found in beer, particularly guanosine, may favor hyperuricemia and the risk of gout [50,56]. In fact, no significant differences were found in uric acid plasma concentrations after the consumption of regular beer and freeze-dried beer, the last one with no ethanol, meaning that the uric acid plasma concentration depends mainly on the beer purine content [33]. In summary, gout development is closely related to hyperuricemia that can result from a uric acid overproduction or underexcretion. Since uric acid is the end- product of purine catabolism, the latter compounds are considered inductors of these diseases, being the reason behind the restriction of purine-rich foods and beverages. To avoid these restrictive approaches and clinical consequences and burdens, efficient techniques to remove these metabolites from foods and beverages should be developed.

## 3. Removal of Purine Compounds from Beer

Beer consumption represents a risk factor of gout, mainly due to its purine content; therefore, a reduction on the intake of these molecules is considered an effective practice to prevent and manage the disease [42,56,84,92]. In this sense, it is of extreme importance to develop techniques that allow the removal of purine compounds from food products. This possibility will assist hyperuricemic and gouty patients to achieve a proper management of the disease, avoiding the common restrictive dietary methods, while enabling them to maintain serum uric acid concentrations within the reference value and reduce clinical manifestations. Accordingly, efforts have been carried out to reduce the purine content in food items, being focused on enzymatic degradation or on adsorption methods, using a variety of adsorbent materials, which are overviewed below.

### 3.1. Enzymatic, Biological and Processing Methods

The application of enzymes or microorganisms in food products is an old process, experiencing a significant increase in recent years [93,94]. Despite their relevance, few methods were, however, reported by envisaging the reduction of the total purines content of malt-derived fermented beverages and foods [95,96,97]. Given the limited number of works in this field, those applied to food items are also reviewed, and they could be of value to design strategies to remove purine compounds from beer.

Shibano et al. [96] disclosed a brewing process to obtain beer with a reduced purine content. A nucleoside phosphorylase isolated from calf spleen was activated and applied to wort to allow the catabolism of purine nucleosides into their bases, which were further assimilated by yeast during fermentation. In wort without enzymatic treatment, it was verified a decrease on the amount of purine bases (e.g., adenine, guanine and xanthine), from 70 to 10 mg L^−1^, during the initial stage of fermentation, whereas no changes were detected on the amount of purine nucleosides (e.g., adenosine, guanosine and inosine; approximately 90 mg L^−1^). This proves that purine bases are uptake and assimilated by yeast during fermentation. However, after treating wort with the isolated enzyme, it was found that a decrease on the purine nucleosides contents, whereas an increase in the concentrations of bases was detected. A nucleoside decomposition ratio of around 60% was determined, proving the enzyme effectiveness on the decomposition of nucleosides. Since it was proved that purine bases are further assimilated by yeasts, the same compounds resultant from purine nucleosides decomposition could be also uptake and used, leading to a beer with a low purine content. This method is, however, dependent on the degradation rate of purine nucleosides and on the assimilation rate of the total purine bases and nucleosides initially present in wort [98].

Mahor et al. [99] demonstrated the possible role of a purine nucleoside phosphorylase (PNP) from *Kluyveromyces lactis* (KlacPNP), an enzyme involved in the purine catabolism, and its variant KlacPNPN256E, in catalyzing a crucial reaction that leads to a reduction on the purine contents in beer. According to the results, after treating beer with these enzymes, a significant conversion of inosine into hypoxanthine by KlacPNP, as well as inosine to hypoxanthine and adenosine into adenine by KlacPNPN256E, occurred. These findings suggest the applicability of KlacPNP and respective variants on the crucial steps that contribute to decrease the purine contents in beer. Moreover, Mahor et al. [65] used adenine deaminase and guanine deaminase, involved in the conversion of adenine to hypoxanthine and guanine to xanthine, respectively, of *Kluyveromyces lactis*, to reduce the purines content in beer. A reduction on adenine contents of 66–67% and a decrease of guanine concentration from 68.8 µmol L^−1^ to residual values were found. Furthermore, when adenine and guanine deaminases were combined with other important degrading enzymes and applied in beer, a reduction of the total purine content was verified.

Jankowska et al. [95] reviewed the role of a novel enzymatic approach, using *Arxula adeninivorans* endogenous and recombinant purine degradative enzymes, in the manufacture of food products with low purine compounds content. This enzymatic system is composed of four purine-degrading enzymes, namely adenine deaminase, guanine deaminase, xanthine oxidoreductase and urate oxidase, that simultaneously catabolize purines into the water-soluble compound 5-hydroxyisourate and preventing uric acid accumulation. This enzymatic mixture was applied in a rolled fillet of ham, and it was verified that, after inoculation, adenine, hypoxanthine, xanthine, guanine, and uric acid contents were reduced. The authors [100] used a purified recombinant *Arxula adeninivorans*, capable to produce high amounts of adenine deaminase to achieve a beef broth with lower purine contents. This enzymatic methodology was able to reduce adenine contents from 70.4 to 0.4 mg L^−1^, paving the way for its use to reduce the production of uric acid. Moreover, Jankowska et al. [101] used *A. adeninivorans*, which is able to produce xanthine oxidoreductase to obtain food products with a reduced xanthine and hypoxanthine contents. It was found that there was a reduction from 169.24 to 39.59 mg L^−1^ in xanthine concentrations, along with a decrease from 171.56 to 52.22 mg L^−1^ in hypoxanthine contents. Although this mixture of enzymes was only applied to reduce the purine contents in food items, its use in fermented malt beverages, such as beer, could also be seen as a promising possibility.

Chen et al. [97] proposed a method to reduce purine content of an edible material using specific microorganisms capable of digesting these molecules, followed by their removal through conventional separations methods (e.g., centrifugation and filtration). The microorganisms were selected from *Aspergillus Oryzae* ATCC 11 493, *Aspergillus Oryzae* ATCC 26831, *Aspergillus Oryzae* ATCC 44193, *Rhizopus oryzae* ATCC 52362 and combinations of both. This biological method was applied in water extracts of mushrooms and soy-bean milk, in which adenine, guanine, hypoxanthine and xanthine contents were reduced after treatment. Moreover, by using *Aspergillus Oryzae* ATCC 26831 (1.3 × 106 spore/mL), a reduction of 84, 61 and 97% of the total purine content in three distinct mushroom water extracts, respectively, was verified after 48 h, whereas, by using the same microorganism and *Aspergillus Oryzae* ATCC 44193 (1.3 × 106 spore/mL), a reduction of 15.24 and 39.93% in soy-bean milk, respectively, was verified after 16 h. These findings suggest the effectiveness of these microorganisms in reducing the total purine content of edible materials. This biological method could be also applied to reduce purine content of alcoholic beverages, such as beer, and as suggested by the authors.

Li et al. [102] described a chemical processing method, involving the use of allicin to reduce the purine content in turbot fish during cooking. It was demonstrated that, during the soaking phase, allicin contributes to decrease the concentration of purines by slightly increasing xanthine oxidase activity that leads to the conversion of hypoxanthine to xanthine. According to the obtained results, a purine reduction of 70.45% was achieved, proving the applicability of this method to reduce purine contents in food products. Li et al. [103], by employing a method to extract purine compounds (e.g., adenine, hypoxanthine, guanine and xanthine) from marine fish, discovered that a boiling process significantly reduces the purine content in this food item. Hypoxanthine registered higher reductions on its contents of 70.24 and 52.57% in dorsal and abdominal muscles, respectively. It was also pointed out that this purines reduction may be due to their transference to the cooking liquid during boiling.

Overall, and although scarcely studied, enzymatic and biological methods allow for the reduction of purine compounds’ content in foods and beverages, including beer. Nevertheless, the applicability of enzymatic methodologies in the food industry brings out additional challenges, such as the difficulty of enzymes to preserve their activity under conditions compatible with the food-storage conditions for long periods, and for which further research in this field is highly recommended.

### 3.2. Adsorption Methods

Given the limitations observed for enzymatic and biological methods, it is imperative to develop other methodologies that could allow an efficient removal of purine compounds from foods and beverages. This will allow the manufacture of products with low purine contents, enabling their consumption by hyperuricemic and gouty patients and the decrease of the risk to develop these diseases. Adsorption methods can be an efficient alternative towards these goals. Despite their potential, there are still few reports focused on the removal of purine compounds from matrices such as beer and wort, and these are dominated by adsorption processes that use carbon-based materials, namely activated carbon or charcoal [98,104,105]. These works, as well as the previously mentioned enzymatic degradation methodologies for purines removal, are summarized in Table 5.

Activated carbon or charcoal is a common adsorbent material due to its several advantages, i.e., high surface area, high adsorption capacity, rapid adsorption kinetics and relative easiness in being regenerated [106]. For instance, Buday et al. [104] reported the use of activated carbon to recover concentrated nucleic acid derivatives, such as guanine or guanosine, from biological samples, for instance, wort and beer. Fujino et al. [98] proposed a method to obtain malt fermented beverage with a reduced purine concentration, in which these molecules were efficiently and selectively removed from wort or fermentation solution by using activated charcoal as an adsorbent material. It was found that an activated carbon with an average pore diameter of 1–3.5 nm and occurring as a fine powder is more effective in removing purine compounds (adenosine, guanosine, and guanine) from aqueous solutions. However, when applied to beer and low-malt beer samples, it was found that an activated charcoal with an average pore diameter of 1.8 nm, added in 500 mg/100 mL, is more effective, with an adsorption capacity for purine compounds ≥90%. The selectivity for the material for these molecules was also evaluated, with no alterations found in the contents of other compounds, such as ethanol and amino acids, proving the high selectivity and efficiency of the material to remove purines from beer and low-malt beer. Nevertheless, a decolorization event could occur in the resultant fermented malt beverages after treatment with activated charcoal.

Shibata et al. [105] used activated carbon, prepared from brewing waste material, i.e., beer lees, to remove purine compounds from beer. A variety of beer lees-based activated carbons were prepared (e.g., LPN37, C950H1(2), C940H1.5(3) and C960H1.5(3)), and their adsorption capacity towards adenine, adenosine and AMP was evaluated and compared to those of a commercial activated carbon. It was found that the prepared beer lees-based activated carbon materials can remove the target molecules with an almost equal removal efficiency to that of the commercial activated carbon. It was also concluded that the total purine bases’ contents decrease with the increase in the amount of activated carbon employed. When 3 g/100 L beer of C940H1.5(3) or C960H1.5(3) was used, an almost 100% removal of the total amount of purine bases was accomplished; however, when the same amount of LPN37 or C950H1(2) was applied, this content decreases to 10–20 mg/L. These results shown that C940H1.5(3) and C960H1.5(3) present a higher adsorption capacity for purine compounds in beer. Nevertheless, there are some disadvantages associated with the use of this kind of materials. Due to their molecular sieve behavior, the adsorption ability decreases for relatively small purines (e.g., adenine), while for relatively large purines (e.g., AMP) the adsorption ability increases. Furthermore, these materials displayed low selectivity towards purines, since other molecules, such as amino acids, flavor -related compounds and essential nutrients, were simultaneously removed.

Given the lack of work focused on the removal of purine compounds from beer by adsorption methods, studies involving the adsorption of purines and related molecules from other matrices are also here overviewed, paving the way for its applicability in beer. To get solutions to better manage gout disease, several researchers developed various adsorbent materials able to remove purines and related compounds. Among them, microporous polymer granule [107], zinc oxide nanoparticles loaded on activated carbon [106] and polyethyleneimine/SiO_2_ [108] were developed, and their potential to remove uric acid from blood sample, preventing the excessive serum uric acid content, was tested. For instance, the adsorption of uric acid onto zinc oxide nanoparticles loaded on activated carbon (ZnO-NP-AC) was studied, and the efficacy of this adsorbent was proven [106]. A high adsorption capacity of 345.8 mg/g for the removal of uric acid was achieved. This finding suggests the applicability of activated carbon materials to adsorb and remove uric acid, which may prevent an excess of this metabolite. Additionally, Liu et al. [109] studied the impact of a pitch-based spherical activated carbon, modified by chemical vapor deposition of NH_3_, on uric acid adsorption properties. It was found that its adsorption occurs as a spontaneous, endothermic and irreversible process, thus suggesting its possible application to remove uric acid from blood samples. Since these adsorbent materials were able to efficiently remove uric acid from blood, resolving gout disease in a more evolved stage, i.e., after uric acid formation during purine catabolism, their applicability to remove purine molecules from beer could also be envisioned. It was also reported the use of dextrans [110] and phyllosilicates [111] as adsorbent materials to remove purines compounds and nucleic acids from aqueous solutions, respectively. Since beer is an aqueous solution presenting a panoply of components, including purine compounds, the applicability of these adsorbent materials to remove these target molecules from such matrices could also be useful and envisioned for this propose.

Given the features exhibited by activated charcoal materials, Tsurushima et al. [112] proposed an adsorption method based on activated carbon to efficiently separate nucleotides and nucleosides from a solution containing both compounds. After adsorption, nucleotides were successfully eluted with an aqueous solution of an alkali metal hydroxide, proving the ability of activated carbon to adsorb these target compounds. Icenhour et al. [113] proposed an adsorption procedure, using activated charcoal coated with polyvinylpyrrolidone, dextran or coconut flours, to extract nucleic acids, namely DNA and RNA, from complex matrices, such as stool and water samples.

Beyond activated carbons, other novel materials have been proposed for the adsorption of purine compounds and relatives, such as carbon nanotubes (CNTs) and graphene-based materials [114,115]. CNTs, discovered in the 1960s, correspond to hollow cylindrical sheets of hexagonal carbon atoms networks, being considered a metallic or a semi conductive material [114]. They exhibit a high surface area, accompanied by a small diameter and high curvature, allowing the establishment of effective interactions with target compounds, mainly through van der Waals forces, π–π stacking and hydrophobic interactions. Due to their advantages, some studies have been reported that proves the adsorption of nucleic acids and purine compounds on CNTs surface, although the majority of them focused on the evaluation of the interactions involved on the adsorption process [116,117]. For instance, Yaroslav et al. [117] studied the adsorption of adenine, thymine and their radicals on the surface of metallic and semiconducting single-wall carbon nanotubes. It was found that these molecules are physisorbed, mainly due to the interactions of their π-orbitals with the π-orbitals of the adsorbent. Moreover, Nandy et al. [116] studied the adsorption of nucleic acids, namely single- and double-stranded DNA (ssDNA and dsDNA, respectively) on CNTs, demonstrating that single stranded DNA (ssDNA) strongly adsorbs and wraps around CNT surface, with almost all nucleobases interacting with the material through van der Waals forces. The same group of researchers [116] evaluated the adsorption of the same nucleic acid (ssDNA and dsDNA) on the surface of a graphene material.

Graphene emerged in 2004 and, since then, aroused great interest within the scientific community [115,118]. In its pristine form, graphene corresponds to a two-dimensional (2D) material, formed by a thin layer of carbon atoms arranged in a sp2-bonded aromatic structure with a one-atom thickness, also known as honeycomb crystal structure [114,119]. Graphene presents unique physical, chemical and thermal properties, being considered an ideal material for a diversity of applications [114,120]. Within its panoply of attributes, graphene exhibits a high surface area, which is related to its high capacity to adsorb organic molecules [120,121,122,123]. Based on these features, Nandy et al. [116] showed that dsDNA can be adsorbed on the surface of graphene, which is mainly due to non-covalent interactions, particularly π–π stacking. It was found that the binding affinity of the target molecule to graphene is higher, comparing to those obtained in CNTs. This can be explained by the advantageous properties exhibited by graphene materials, such as their high surface area and their easy surface modification [119]. Moreover, Antony et al. [124] studied the process and interactions energies involved in the adsorption of DNA nucleobases, including guanine (G), adenine (A), cytosine (C) and Thymine (T) on graphene, by dispersion-corrected density functional theory (DFT-D). It was found a binding crescent sequence as follows: G > A > T > C. Similarly, Sowerby et al. [125] used a single solute adsorption isotherm method to study the adsorption process of the same DNA nucleobases onto graphite-water interface, and it was found a similar adsorption strength crescent sequence (G > A > T > C). On the other hand, Gowtham et al. [126] evaluated, through a plane-wave pseudo potential approach within the local density approximation (LDA) of DFT, the interaction energies involved in the adsorption of the same DNA nucleobases onto graphene, and they found an energy-binding sequence following the order G > A~T~C. Finally, Varghese et al. [127] studied the adsorption process of the same DNA nucleobases onto graphene, and it was found that the binding energies involved are generally weak, exhibiting the sequence G > A > C~T. These findings prove that DNA nucleobases can adsorb onto graphene materials, the binding energies involved are generally weak, and guanine can stablish a stronger interaction with the material when compared to the other nucleobases.

Nandy et al. [116] studied the adsorption and interactions involved of single and double stranded DNA (ssDNA and dsDNA, respectively) on a variety of materials, including CNTs, dendrimers and graphene, and it was found that dsDNA macromolecules adsorbs better onto graphene. Liu et al. [128] studied the adsorption of DNA onto gold nanoparticles and graphene oxide (GO), being found that the GO heterogeneous surface (due to the presence of hydrophobic regions) is favorable for DNA adsorption. Moreover, Lee et al. [129] demonstrated the adsorption of DNA onto graphene, and it was found that beside π–π stacking, other interactions can take place, leading to an interfacial dipole interaction between nucleobases and graphene. These findings prove not only that DNA, and its derivatives have the capacity of being adsorbed onto graphene materials, but also that this process is mainly driven by π–π stacking interactions.

The works regarding adsorption methods of purine and close compounds using a diversity of adsorbent materials are compiled in Table 6. It is important to reinforce that, although a majority of the exposed adsorption works focus on the study of the process and interactions involved, the high capability of these materials to adsorb purine molecules and close compounds was shown. These findings are highly important in the sense that they could pave the way for the application of these materials in the food industry to remove purine compounds from food items, and in particular, from beer.

## 4. Conclusions

Beer is composed of a panoply of important compounds, and among those compounds are purines. After being ingested by humans, purines can be converted into uric acid, increasing its serum level, which may lead to hyperuricemia and gout. To assure a proper management of this disease, physicians usually recommend restrictive dietary measures, such as avoiding the consumption of beer. Therefore, it is of high relevance to develop efficient methods to remove purine compounds from alcoholic beverages.

Several methods were reported while envisaging the removal of purine compounds from aqueous solutions and beer, including enzymatic, biological and adsorption methods. Considering enzymatic and biological methods, most of them present some drawbacks that could possibly interfere with their application to remove purine compounds from food products, such as the degradation rate and enzyme compatibility with food storage conditions. In this sense, adsorption methods can be seen as alternative promising approaches. Despite their relevance, few works are still focused on the application of adsorption techniques to remove purine compounds from wort or beer samples, envisaging the manufacture of a final product with a low purine content, and these were carried out by employing materials such as activated carbon or charcoal. Nevertheless, there is a plethora of other materials reported for the removal of uric acid from aqueous model solutions and other samples, including 2-hydroxyethyl methacrylate and ethyleneglycol dimethacrylate, zinc oxide nanoparticles loaded on activated carbon, pitch-based spherical activated carbon (PSAC) modified by CVD of NH_3_, and polyethyleneimine/SiO_2_. These materials aim to reduce the risk of developing gout in a more evolved phase, i.e., after uric acid being formed from purines. Based on their high adsorption performance, these materials should be investigated as well for the removal of purine compounds from beverages, including beer. Other adsorbent materials, such as graphene and related materials, carbon nanotubes and dextrans, were applied to adsorb nucleic acids. These studies were mainly focused on the evaluation of the interactions involved, additionally showing their high capacity to adsorb these molecules, and, as such, their high potential to be applied in the removal of purine compounds.

Based on this review, we have identified that more efforts need to be carried out by researchers to develop efficient and selective adsorbent materials that could be applied to remove purine compounds from beer. By ensuring the selective removal of purine compounds from this beverage, beer can be consumed by hyperuricemic and gouty patients, thus avoiding restrictive dietary measures, while decreasing the healthcare-related economic burden.

## Figures and Tables

**Table 1:** Chemical composition of regular beer (information provided in the literature [17,18,28].

	Concentration	Source
Water	90%	-
Ethanol	20,000–80,000 mg L^−1^	Yeast, malt
Carbon Dioxide	3500–4500 mg L^−1^	Yeast, malt
Carbohydrates	3.3–4.4%	Malt
Inorganic salts	500–2000 mg L^−1^	Water, malt
Total nitrogen compounds	300–1000 mg L^−1^	Yeast, malt
Organic acids	200–500 mg L^−1^	Yeast, malt
Higher alcohols	60–100 mg L^−1^	Yeast, malt
Aldehydes	10–20 mg L^−1^	Yeast, hops
Esters	60–80 mg L^−1^	Yeast, malt, hops
Sulphur compounds	1–10 mg L^−1^	Yeast, malt, hops
Hop derivatives	20–60 mg L^−1^	Hops
Complex B vitamins	5–10 mg L^−1^	Yeast, malt

**Table 2:** Content of the most commonly found purine compounds in beer [33].

	Concentration (µmol L^−1^) *
Guanosine	174 ± 16.1
Xanthine	58.5 ± 3.6
Guanine	42.4 ± 3.6
Adenosine	42.1 ± 7.9
Inosine	20.3 ± 2.5
Hypoxanthine	17.5 ± 1.5
Adenine	17.2 ± 2.3

* All contents are represented as mean (*n* = 3) ± SD.

**Table 3:** Comparison of the total purine content between alcoholic beverages [46].

Alcoholic Beverage	Total Purine Content (µmol L^−1^) *
Beer	13.3–1145.7
Regular	225.0–580.2
Low-malt	193.4–267.9
Low-malt and low-purine	13.3
Local	312.0–1145.7
Low-alcohol	194.8–898.3
Spirits	0.7–26.4
Other liquors	13.1–818.3
Sake	82.1–110.4
Shoku-shu	537.4–818.3
Ume liqueur	13.1
Beer-flavored beverage	73.5–157.4

* The total purines content was determined by HPLC.

**Table 4:** Gout prevalence at a global scale (data given in the studies from References [61,79,80,81,82,83]).

Country	Methodology	Year	Prevalence
USA	Third National Health and Nutrition Examination Survey (NHANES-III) 5467 participants/adults; gout definition: self-reported gout	2007–2016	3.9% (9.2 million); men: 5.2% (5.9 million); women: 2.7% (3.3 million)
South Korea	National Health Claims Database for specialized care; all ages; gout definition: physician-diagnosed	2007–2015	2007: 3.49/1000 individuals 2015: 7.58/1000 individuals 2025 perspective: increment of 1.66%
Australia	National Database of medical records; ≥18 years; Gout definition: diagnosis in medical records	2013–2016	1.6%
UK	Clinical Practice Research Datalink	1997–2012	2.49%; increment of 63.9% since 1997
Portugal	Randomly selected 10,661 adult participants; ≥18 years; Gout definition: ACR 1977 criteria	2011–2013	1.3%; men: 2.6%; women: 0.96%

**Table 5:** Removal-based methodologies of purine compounds from beer [65,96,99,104,105].

Target	Removal Agent	Method	Matrix	Application
Adenine, guanine, xanthine, adenosine, guanosine and inosine	Nucleoside phosphorylase isolated from calf spleen	Enzymatic degradation	Wort	Brewing process for the manufacture of a beer reduced in purines
Inosine and hypoxanthine	Purine nucleoside phosphosphorylase from *K. lactis* (KlacPNP) and KlacPNP^256E^ variant	Enzymatic degradation	Beer	Reduction of the purine content of beer
Adenine and guanine	Recombinant adenine and guanine deaminases of *K. lactis*	Enzymatic degradation	Beer	Reduction of the purine content of beer
Nucleic acid derivatives (e.g., guanine and guanosine)	Activated carbon	Adsorption	Wort and beer	Recover and concentration of nucleic acids
Adenosine, guanosine and guanine	Activated charcoal prepared with beer lees	Adsorption	Beer and low-malt beer	Production of a malt fermented beverage with reduced purine content

**Table 6:** Adsorption of purines and close compounds.

Target	Matrix	Adsorbent
Uric acid	Aqueous solutions	2-hydroxyethyl methacrylate and ethyleneglycol dimethacrylate in the shape of granules
Uric acid	Aqueous solutions	Zinc oxide nanoparticles loaded on activated carbon
Uric acid	Aqueous solutions	Pitch-based spherical activated carbon (PSAC) modified by CVD of NH_3_
Uric acid	Aqueous solutions	Polyethyleneimine/SiO_2_
DNA and RNA	-	Activated carbon coated with polyvinylpyrrolidone, dextran or coconut flours
DNA nucleobases (guanine, adenine, cytosine and thymine)	-	Graphene
DNA nucleobases (guanine, adenine, cytosine and thymine)	-	Carbon nanotubes, dendrimers and graphene
DNA nucleobases (guanine, adenine, cytosine and thymine)	Aqueous solutions	Graphene
Adenine, adenosine and AMP	Aqueous solutions and beer	Activated carbon derived from beer lees
DNA nucleobases (guanine, adenine, cytosine and thymine)	-	Graphene
DNA nucleobases (guanine, adenine, cytosine and thymine)	-	Graphene
Adenine, thymine and radicals		Single-wall carbon nanotubes
Adenosine, guanosine and guanine	Wort, beer and low-malt beers	Activated charcoal
DNA nucleobases (guanine, adenine, cytosine and thymine)		Graphite
Nucleotides and nucleosides		Activated carbon
Adenine and xanthine	Aqueous solutions	Dextran T40 and Sephadex G-10

## Data Availability

Not applicable.
